# Specialized Staff for the Care of People with Parkinson’s Disease in Germany: An Overview

**DOI:** 10.3390/jcm9082581

**Published:** 2020-08-10

**Authors:** Tino Prell, Frank Siebecker, Michael Lorrain, Lars Tönges, Tobias Warnecke, Jochen Klucken, Ingmar Wellach, Carsten Buhmann, Martin Wolz, Stefan Lorenzl, Heinz Herbst, Carsten Eggers, Tobias Mai

**Affiliations:** 1 Department of Neurology, Jena University Hospital, 07740 Jena, Germany; 2 Center for Healthy Ageing, Jena University Hospital, 07740 Jena, Germany; 3 Praxis Neurologie, 48291 Telgte, Germany; fs@neurologie-telgte.de; 4 Neuroärzte Gerresheim-Pempelfort, 40625 Düsseldorf, Germany; dr.lorrain@volggerconsult.de; 5 Department of Neurology, St. Josef-Hospital, Ruhr-University Bochum, 44801 Bochum, Germany; lars.toenges@rub.de; 6 Department of Neurology, University of Muenster, 48149 Münster, Germany; Tobias.Warnecke@ukmuenster.de; 7 Department of Molecular Neurology, Universitätsklinikum Erlangen, Schwabachanlage 6, 91054 Erlangen Neurology, Ev. Amalie Sieveking Hospital, 22359 Hamburg, Germany; Jochen.Klucken@uk-erlangen.de; 8 AG Digital Health Pathways, Fraunhofer Institute for Integrated Circuits, Am Wolfsmantel 33, 91058 Erlangen, Germany; 9 Münster Medical Center Hamburg-Eppendorf, 20246 Hamburg, Germany; 10 Department of Neurology, Ev. Amalie Sieveking Hospital, 22359 Hamburg, Germany; ingmar.wellach@immanuelalbertinen.de; 11 Office for Neurology and Psychiatry Hamburg Walddörfer, 22359 Hamburg, Germany; 12 Department of Neurology, University Medical Center Hamburg-Eppendorf, 20246 Hamburg, Germany; buhmann@uke.uni-hamburg.de; 13 Department of Neurology, Elblandklinikum Meißen, 01662 Meißen, Germany; Martin.Wolz@elblandkliniken.de; 14 Professorship for Palliative Care, Paracelsus Medical University, 5020 Salzburg, Austria; stefan.lorenzl@pmu.ac.at; 15 Department of Palliative Medicine, Ludwig-Maximilians-University Munich, 81377 Munich, Germany; 16 Department of Neurology, Klinikum Agatharied, 83734 Hausham, Germany; 17 Neurozentrum Sophienstrasse, 70178 Stuttgart, Germany; heinz.herbst@t-online.de; 18 Department of Neurology, University Hospital Marburg, 35037 Marburg, Germany; Carsten.Eggers@uk-gm.de; 19 Department of Nursing, University Hospital Frankfurt, Goethe University, 60590 Frankfurt, Germany; tobias.mai@kgu.de

**Keywords:** networks, multimodal complex treatment, day clinic, advanced care planning

## Abstract

Access to specialized care is essential for people with Parkinson´s disease (PD). Given the growing number of people with PD and the lack of general practitioners and neurologists, particularly in rural areas in Germany, specialized PD staff (PDS), such as PD nurse specialists and Parkinson Assistants (PASS), will play an increasingly important role in the care of people with PD over the coming years. PDS have several tasks, such as having a role as an educator or adviser for other health professionals or an advocate for people with PD to represent and justify their needs. PD nurse specialists have been established for a long time in the Netherlands, England, the USA, and Scandinavia. In contrast, in Germany, distinct PDS models and projects have been established. However, these projects and models show substantial heterogeneity in terms of access requirements, education, theoretical and practical skills, principal workplace (inpatient vs. outpatient), and reimbursement. This review provides an overview of the existing forms and regional models for PDS in Germany. PDS reimbursement concepts must be established that will foster an implementation throughout Germany. Additionally, development of professional roles in nursing and more specialized care in Germany is needed.

## 1. Background

Parkinson’s disease (PD) is a common neurodegenerative disorder characterized by motor symptoms such as tremor, rigidity, bradykinesia, and a plethora of nonmotor symptoms (NMS). The appearance and severity of motor symptoms and NMS vary throughout the disease course and contribute to different degrees of functional impairment and reduced quality of life [1]. Therefore, access to different levels of care is essential for the increasing number of people with PD [2]. An important factor here is specialized outpatient and inpatient medical care as well as the well-coordinated trans-sectoral transition from hospitalized inpatient to outpatient to homecare, and vice versa [3]. Patients with PD need continuous specialized outpatient care, which can be supplemented by more intensive inpatient treatment (such as PD multimodal complex treatment (PD-MCT), PD day clinic, telemedicine) if necessary [4,5,6]. Frequently, PD patients also must consult emergency care at local hospitals [7,8]. Reasons for hospital admission are PD-related symptoms [9] as well as infections, gastrointestinal disorders, falls, neuropsychiatric, and other health problems. In hospitals and wards without specialized neurological knowledge, there is an increased risk of discontinuation, inappropriate change of medication, or use of inappropriate or contraindicated drugs. Such can lead to the worsening of motor function, falls, and delirium or comorbidity with all its known secondary complications, especially in the elderly with PD. Such complications are often associated with a higher risk of long term care in nursing homes [10].

People with PD need specially structured and cooperative therapy, education, and care concepts both in the short and long run. Qualified PD-specific nonphysician staff (PDS), such as PD nurse specialists (PD nurse) and Parkinson Assistants (PASS), can play an important role. Among others, PDS can fulfill a role as an educator or adviser for other health professionals in hospitals and be an advocate for the people with PD to represent and justify their needs. Such is also relevant given the lack of general practitioners (GP) and neurologists in rural areas [11]. Experiences in other countries (e.g., the Netherlands, England) show that PD nurses can provide a large part of care and treatment [12,13,14,15]. Given the growing number of PD patients, it is difficult to provide comprehensive care solely through physicians and nurses alone. PD nurses and other PDS could engage in routine support and could spend more time on more complex cases [16]. Moreover, in Germany, the majority of PD nurses work in hospitals, so there is a lack of qualified specialists, especially in outpatient care and in nursing homes. However, the German S3 PD guideline recommends that every PD patient should have access to PD nurses [17]. Over the disease course, the caregiver burden is often high, and specialized staff in outpatient care should play a key role in addressing potential problems. This article provides an overview of the existing forms and regional models of PDS in Germany.

## 2. Field of Activities and Responsibilities

The expenditure of time needed to care for people with PD has increased enormously recently. Such is based on a higher complexity of revised clinical diagnostic criteria, new technical diagnostic methods, and individualized therapeutic care considering the growing known spectrum of PD symptoms defining different motor and nonmotor subtypes of PD [18]. Specialized, holistic, lifelong care of people with PD requires practices that focus on the disease-specific needs of the individual, their family members, and caregivers. However, the treatment should not only account for the individual course of the disease with its plethora of motor symptoms and NMS. The often far-reaching psychosocial problems must also be taken into consideration (e.g., coping with the diagnosis, fear of uncertainty about the individual course of the disease, changes in family structures, job and pension problems, need for care). Given the high prevalence of cognitive impairment and dementia in PD, these symptoms also need special attention. Patients, relatives and caregivers need advice how to cope with cognitive decline and what kind of therapies and strategies are available. The PDS can provide assistance and support for many aspects and challenges during the PD course. Such assistance can maintain and improve the quality of life for people with PD, provide support and education to patients and healthcare professionals, and support and provide a seamless service throughout the disease trajectory [19]. Especially the complex aspects of palliative care at the late stage of PD require a well-positioned interdisciplinary team of PD specialists.

Discontinued care and lack of self-management are frequently associated with improper handling of PD medication. Nonadherence to medication is a significant issue in PD. It results in frequent hospitalizations, reduced quality of life, and causes a financial burden for the health system [20,21,22,23,24,25,26]. There are various reasons why people do not or cannot follow the given recommendations and instructions for the prescribed treatment. PDS can improve knowledge about medication or identify reasons for nonadherence. In summary, PDS can [12,13,14,27,28,29,30,31]:

- Help to cope with PD, answer frequent and everyday questions and give advice where doctors lack the necessary time: Typical questions are for example “What do I have to pay attention to with the medication?”, “Can I go on holiday?”, or “Can I do sports?”
- Use assessments to identify and monitor symptoms, side effects, and family problems
- Advise on the motor and NMS and complications.
- Counsel relatives and monitor their burden
- Teach other health and social care professionals (e.g., for handling pumps or deep brain stimulation (DBS))
- Inform comprehensively about therapy options, self-help groups or socio-medical aspects, such as applying for care levels or certificates for severely disabled persons
- Help to improve adherence to medication
- Assist in the initiation and adjustment of continuous therapies or take over most of them independently
- Support in making the PD diagnosis (e.g., performing an L-dopa or apomorphine challenge test)
- Make referrals to other professionals such as speech and language therapists, occupational therapists, physiotherapists or social workers and support networking between different therapeutic players
- Assist in advanced care planning (ACP)

In the UK, distinct competency levels for PD nurses were defined, ranging from a registered competent nurse (Level 5 of the Career Framework for Health), experienced specialist nurse (Level 6), expert specialist nurse (Level 7) to consultant nurse (Level 8) [19]. In contrast, in Germany officially certified and reimbursed models for PD do not exist. Distinct models and projects show a relevant heterogeneity in terms of access requirements, education, theoretical and practical skills, and principal workplace (inpatient vs. outpatient). In the following, we present established PDS in the German healthcare system.

## 3. Parkinson’s Disease Nurse Specialist (PD Nurse)

Specialized nurses for patients with PD (hereinafter, PD nurse) have been available for over 40 years in several countries, including England, the USA, and the Scandinavian countries. PD nurses are acknowledged and valued as part of the multi-professional PD team [30]. The work tasks for PD nurses as mentioned above vary between different workplaces. However, they can include case management tasks, care of patients with complex therapies such as pen/pump therapies and DBS, scoring and assessments, and clinical research tasks. The experience with this specialized nurse function has been consistently positive. It shows improved care for patients with PD, improved productivity and quality of clinical research, as well as improved job satisfaction of the participating employees [12,13,27,29]. For many patients, the PD nurse is the primary link to medical care as most PD specialist nurses have an open phone line [14].

The German Parkinson Society (DPG), the German Parkinson Association (dPV), the Competence Network Parkinson (KNP) and the Association of Parkinson Nurses and Assistants (VPNA e.V.) have developed an education curriculum representing the standard for PD nurses in Germany. The first training course started in 2007. Applicants should be qualified health and nursing professionals and have a minimum of two years of professional experience in acute neurological departments or PD hospitals (Table 1). Training comprises four days of theoretical training and two weeks of work shadowing, spread over a year. Knowledge of the disease and the treatment are crucial for the work of a PD nurse [28,31]. The duties of the PD nurse also includes practical tasks (e.g., examine and evaluate the patient’s health and motor functions, collect blood samples, adjust the settings in DBS and pumps) [12]. They play a vital role in the new concept of Parkinson Day Clinics in Germany [32].

The most reported tasks of PD nurses in Germany are giving information and advice to people with PD and their next of kin in the context of medication and side effects, education and counseling to PD symptoms and specialized therapies (and education of other nursing staff) [13,29]. Additionally, nursing functions include screening and offering prevention, supporting patients and caregivers in psychosocial well-being, care coordination and case management, and palliative care and multidisciplinary collaboration. According to the European Qualification Framework, the more expanded international role of a PD nurse is on level 6 or 7 [13,19]. Most PD nurses have completed specialized modules as part of a master course. In Germany, the PD nurse course is based on level 5 of the German Qualification Framework. It is similar to the registered competent nurse at level 5 of the Career Framework for Health [19]. For example, a qualified training course for PD nurse comprises 30 to 40 credit points. The German qualification course is shorter, and there is no state certificate. Recognition as specialized training in Germany usually requires 720 h or more (e.g., a professional training in critical care). Such may explain the different PD nurse role expansion in Germany and international comparison. As in other countries, counseling and education, information on medication management, educational advertising on PD and training of other professionals are at the center of German PD nurses [29]. German PD nurses also stated that they have not enough time for appropriate care and nursing, just like PD nurses have mentioned in the UK (16). PD nurses have a more extensive caseload than the suggested manageable number of 300 patients [15] and therefore need to be substantially supported.

As a future goal, PD nurses can support palliative outpatient teams when caring for the patient and the relative in the last phase since PD patients often suffer from symptoms unknown to palliative care teams. They might visit the patient together with the team or could be the specialist visiting the patient while a telemedical approach discusses symptom control [33].

## 4. Parkinson Assistant (PASS Concept)

At the end of 2000, the Association for Quality Development in Neurology and Psychiatry (QUANUP e.V) was founded as a joint initiative of the Professional Association of German Neurologists (BVDN) and the Professional Association of German Neurologists (BDN). QUANUP has developed a training program for nonmedical staff in neurological doctor´s practices and neurological units in hospitals to qualify them as PASS. Participants were mainly medical assistants with formerly three-year training (Table 1). Since 2009, QUANUP has regularly conducted these structured advanced training events at several locations throughout Germany. The PASS basic course-advanced training is conducted on two weekends on Friday afternoons and Saturday full-time with a total of 14-course hours per weekend. Between the two weekends, homework with self-study should be completed. The participants of the further training courses will receive a certificate after passing the final examination. The first course discusses essential topics (e.g., occurrence and frequency of the disease, possible causes, and mechanisms of PD). The teaching of the PASS includes knowledge about symptoms and complications of the disease, diagnostic procedures, medication and non drug-related treatment options. Case presentations, role playing, and video demonstrations improve practical skills in addition to theoretical knowledge. Neurologists from PD specialist practices and registered PASS lead the PASS courses. An advance course supplements this basic course after 6–12 months. This course includes the teaching of specialized treatment methods such as handling pumps, pens or DBS, provides knowledge about atypical Parkinson’s syndrome in more detail and allows the discussion of problems that have arisen in daily practice. Furthermore, in all courses, advice on efficient, practical organization is given.

The PASS should be a competent contact person for patients with PD and their relatives in outpatient settings, Parkinson day clinics, and the wards in the hospitals. Through the joint, coordinated deployment of qualified physicians and qualified PASS, work processes in the practices should be made more efficient. PASS cares for people with PD and relatives through short distances and low-threshold contact offers. They provide necessary information for patients and their families and are the hub between patients, neurologists, therapists, and care institutions. Among others, PASS can also be involved in: (1) visits of neurological practice unspecialized in PD, (2) PD-specialized practices (Parkinson’s Practice) [34], and (3) video-supported homecare of patients in a telemedical approach.

For the certificate “Parkinson’s Practice” awarded by the German Parkinson Association (Deutsche Parkinsonvereinigung, dPV), a list of criteria has been compiled [34]. The certificate is issued for three years upon application and after an appropriate review. These criteria include, among others, the continuous care of a minimum number of 120 patients with PD and regular training and counseling offers for patients. Additionally, treating physicians must provide proof of regular training in the field of PD, and at least one practice employee must have completed training as a PASS. Further criteria are the standardized collection and documentation of findings in a PD database and guideline-based treatment concepts.

For PD care in rural areas, telemedicine supplied as outpatient video-supported therapy is a suitable option to provide many PD patients access to specific PD care. Homecare via telemedicine may improve patient satisfaction, increase participation, and adherence to therapy substantially. A low-threshold connection between patients and relatives through PASS in neurological practices is another critical issue. This, for example, can significantly reduce complications due to incorrectly taken medication. A structured approach must be developed for Germany on how televisits should be carried out by PDS. Moreover, a reimbursement of this service by the health insurance companies is required.

## 5. Parkinson Care Specialist (Parkinson Pflegespezialist/In)

The target group for the Parkinson care specialist includes nursing staff from wards and outpatient clinics from nursing homes and outpatient nursing and care services as well as staff from nursing support and advice centers (Table 1). To become a Parkinson care specialist, training for two days in a PD center must be completed. The aim of the training is to provide participants with better knowledge regarding PD and allow participants to correctly and professionally implement medical prescriptions and recommendations from PD nurses, PASS or therapists. Parkinson care specialists support their colleagues and organize the appropriate implementation of prescriptions for therapies and therapeutical settings as well as the appropriate care planning and documentation in their department. Moreover, they are familiar with the unique features and effects of PD drugs, the variety of NMS, nutritional aspects, DBS, and pumps. Advanced courses are offered once a year and should be part of this qualification. VPNA e.V organizes the courses.

## 6. Support of Health Care Assistants in General Practice and Outpatient Clinics

Due to the increasing number of chronically ill people and the increasing shortage of GPs but also of hospital-based care, especially in rural areas [4,35], there are many courses to provide healthcare assistants to support medical doctors. There is a high diversity in these training courses [36] (Table 1). Furthermore, Advanced Nurse Practitioners (ANP) are implemented in hospitals to support PD patients. In the outpatient setting, healthcare assistants are trained to support medical doctors. In the review by Günther and colleagues, many projects are mentioned for primary care (e.g., VERAH, AGnES, EVA, MoNi, and MoPra). These training courses for healthcare assistants should comprise at least 150 h. VERAH relates to care assistants in general practice (Versorgungsassistentin in der Hausarztpraxis) and who can do home visits. They also take over delegated tasks from the physicians and advise patients regarding prevention and other health-related questions. They are working as case managers and wound managers as well. AGnES is an acronym for physician-relieving, community-oriented intervention with E-health support (Arztentlastende, gemeindenahe, E-health-gestützte systemische intervention). This approach focuses on older and chronically ill patients who are living at home. In addition to VERAH, the healthcare assistants assess diagnostic parameters and do standardized health monitoring. The tasks are much broader than in the VERAH project, but they also do no nursing tasks. The nursing process is a statutory duty of qualified nurses; it is a certified and reimbursed service (Education Act, Pflegeberufereformgesetz–PflBRefGe §4). However, it is not easy to maintain this distinction because in real work life, the borders are blurring.

MoNi stands for Model of Lower Saxony (Modell Niedersachsen). It is a similar project to AGnES and VERAH in cooperation with the Ministry for Social Affairs, Women, Family, Health Care and Integration of Lower Saxony and the physician Association of Lower Saxony. MoPra means mobile practice assistant (Mobile Praxisassistentin) and meets the criteria and tasks of AGnES. MoPra is well established in Saxony-Anhalt.

In cooperation with the Association of Statutory Health Insurance Physicians North Rhine-Westphalia, the North Rhine-Westphalian Academy offers in-service training to become a “relieving care assistant” (EVA–Entlastende Versorgungsassistentin). The prerequisite for participation is a qualified professional qualification as a Medical Specialist Assistant or nurse. Moreover, at least three years of professional activity in a family doctor’s practice must be proven. Depending on the professional experience, the total EVA training comprises 170–221 h of theoretical instruction and 20–50 h of practical training. In this course, approximately 30% self-study is integrated on a learning platform. The specialization qualification EVA concludes with a certificate from the Association of Statutory Health Insurance Physicians of North Rhine-Westphalia. The further training aims to give the medical assistant the skills to take over delegable services in outpatient practice. The EVA acquires the competence to take over services in the outpatient practice eligible for delegation. In this way, EVA relieves the physician of the burden of accompanying and supporting patients and their relatives in various tasks relating to the treatment process. The training includes different aspects, such as geriatric syndromes, care and support of oncology and palliative patients, pharmaceutical supply, wound care and wound management, coordination and organization of therapy and social measures, telemedical basics, communication management, medical documentation, and practical training (home visits). Details are given on the homepage of the North Rhine-Westphalian Academy: http://www.akademienordrhein.info/eva-entlastende-versorgungsassistentin.

In terms of PD, the North Rhine-Westphalian Academy offers advanced training to become a “Support Care Assistant in Neurology/Psychiatry (EVA-NP).” This training includes skills required to take over delegated services in specialist neurological and psychiatric practice. EVA-NPs are familiar with many neurological and psychiatric clinical syndromes and disorders and are a qualified contact person for patients. Depending on the professional experience, the complete training for the EVA-NP comprises 197–247 h of theoretical instruction and 20 h of practical training (home visits). Various modules are offered within the theoretical instruction. A PD module (24 lessons) can be taken as part of the optional part.

The tasks in every project/position are quite similar. There is an increasing effort to relieve medical doctors and to support the patients. Many healthcare services delegated to healthcare assistants are appreciated and accepted by patients [37]. The increasing shortage of medical doctors in rural areas is the base of increasing demand for qualified healthcare assistants. Nevertheless, doctors are right in their critical view of whether this need for medical delegation of tasks can be taken on by medical assistants with 200 h training [36]. Such is a crucial aspect, especially in distinction of these qualifications from Physician Assistants courses at Bachelor Level or ANP at master level.

## 7. Community Matrons

Because of the high workload of PD nurses in the UK, they usually work in collaboration with community matrons as generic practitioners [15]. Community Matrons are experienced registered nurses with academic training. They have advanced competencies (e.g., in case management or assessments) [38,39] and perform advanced nursing practice, especially for patients with high risk of hospital admission such as people with complex and chronic conditions. Community Matrons can be seen as the extension role of healthcare assistants—but they operate more autonomously. Their qualification is nursing education and an academic training (e.g., a course of community health nursing). In Germany, the German Nurses Association developed a concept for community health nursing (Deutscher Berufsverband für Pflegeberufe) [38]. In a project funded by the Robert Bosch Foundation and the Agnes-Karll-Corporation in the German Nurses Association, three German universities developed a community health nursing course in 2020. Community Health Nurses in Germany should have their tasks in routine activities. These include health and chronic disease related assessments, ensuring adherence to medication, monitoring of symptoms of chronically ill patients, support on self-management, health promotion and prevention, patient and caregiver education, and counseling. They should manage and coordinate health care services as case managers. The authors focus on the care of patients (e.g., with diabetes or PD).

## 8. Proposal of Core Elements for Future PDS Education in Germany

**Figure 1:**
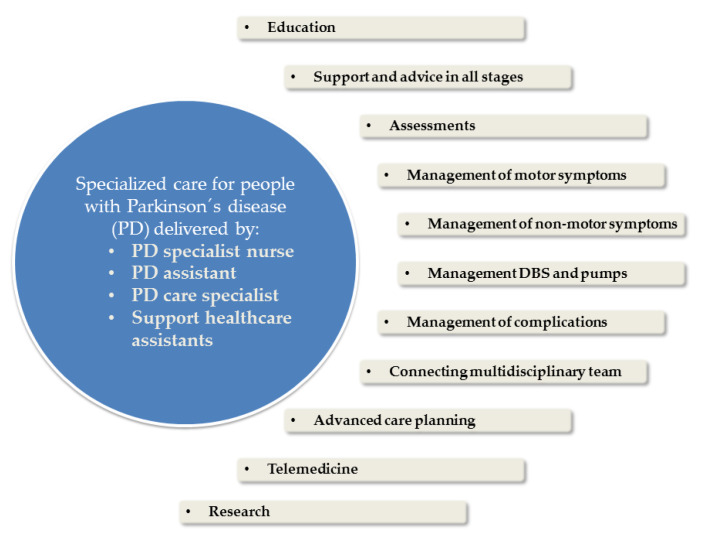
Proposed core elements for future PD staff (PDS) education in Germany.

Today, PDS training in Germany is very heterogeneous and differs in terms of duration, content, and target group. There are courses for health care assistants in general or neurological practices. Within these courses are differences in the specialized view on PD (e.g., PASS with a broader view than AGnES). Additionally, there are existing trainings for nurses. While PD nurses mainly work in specialized hospitals and outpatient clinics, the community health nursing will be operating more in an outpatient setting. In the future, it should be discussed how to integrate more specific PD knowledge in community health nursing qualification, especially for supporting long term care settings and in the community. Moreover, it is essential to develop approaches how these different qualifications could create a functional network for supporting people with PD and their next of kin [40]. In particular, the role of PDS in PD telemedicine needs special attention and standardized procedures. This review was not intended to propose a comprehensive model for future PDS education. However, we propose the core elements given in Figure 1 which is from analysis of the Career Framework for Health [19]. We want to point out that PDS education in Germany needs harmonization, standardization, and reimbursement to improve PD care for all PD patients. In particular, the missing certification and reimbursement are main barriers for the implementation of PDS in outpatient and inpatient structures today.

## 9. Concluding Remarks

Due to the increasing complexity of PD therapy and the availability of specialized therapies for different stages of the disease, an optimized PD treatment requires expert and multi-professional care. However, for many PD patients in Germany, care is provided only by non-specialized neurological practices or GPs without close exchange with neurologists or specialized university outpatient clinics for movement disorders [34]. PDS are important links between outpatient and inpatient care, physicians and therapists, and PDS transfer specialized PD care into so far nonspecialized neurological practices and hospitals. Unfortunately, there is no institutionalized financial support for PDS training. Moreover, the Association of Statutory Health Insurance Physicians does not support or finance independent activity of PDS in outpatients or inpatient setting, which is already possible in other countries such as Great Britain or the Netherlands.

We also need more research on how PDS can improve patient reported outcome and aspects like management/medication adherence, quality of life, palliative care, and functional status/improving function [41]. PDS still require a delegation by responsible physician. To counteract against the current and increasing shortage of physicians, the professional profile and self-image of the physician will have to change, which does not only apply to the PD care setting [36]. It is expected that the physician will have a leading role in an inter-professional team. To establish these teams and include a high standard of continuous education for PDS, reimbursement concepts must be established that will foster an implementation throughout Germany. Furthermore, the development of professional roles in nursing and specialized care in Germany is needed.

## Figures and Tables

**Table 1:** An overview of the existing forms and regional models of Parkinson´s disease (PD)-specific nonphysician staff.

	Target Group and Prerequisite	Education Curriculum	State Certificate	Setting	Organization
Parkinson’s disease specialist nurse	health and nursing professionals minimum of 2 years of professional experience in acute neurological departments or PD hospitals	4 × 2 days of theoretical training +2 weeks of hospital observation, spread over one year	none	inpatient outpatient	Deutsche Parkinson-Gesellschaft, DPG, Deutsche Parkinsonvereinigung, dPV, Kompetenznetz Parkinson, KNP, Verein der Parkinsonnurses u.–assistenten, VPNA
Parkinson assistant	mainly medical assistants with formerly three-year training	basic course (24 teaching hours) advanced course (1-day workshop)	none	inpatient outpatient	QUANUP e. V.
Parkinson care specialist	nurse	two days training in PD center	none	inpatient outpatient	Verein der Parkinsonnurses u.–assistenten, VPNA
VERAH (Versorgungsassistentin in der Hausarztpraxis)	medical assistant	200 teaching units + internship of 40 units	yes	outpatient (GP)	Deutscher Hausärzteverband
AGnESzwei (Arztentlastende, Gemeindenahe, E-health-gestützte systemische intervention)	nurse or medical assistant	129 theoretical teaching units	yes	outpatient (GP)	Arbeitsgemeinschaft “Innovative Gesundheitsversorgung in Brandenburg” (IGiB)–der KVBB
EVA (Entlastende Versorgungsassistentin)	nurse or medical assistant	at least 300 teaching h	yes	outpatient (GP)	Nordrheinische Akademie
